# ASO Author Reflections: Oxidative Stress as a Predictor of Short-Term Outcome After Oncological Surgery for Gastrointestinal Cancer

**DOI:** 10.1245/s10434-022-11453-z

**Published:** 2022-02-28

**Authors:** M. Leimkühler, A. R. Bourgonje, H. van Goor, M. J. E. Campmans-Kuijpers, G. H. de Bock, B. L. van Leeuwen

**Affiliations:** 1 Department of Surgery, University of Groningen, University Medical Center Groningen, Groningen, The Netherlands; 2 Department of Gastroenterology and Hepatology, University of Groningen, University Medical Center Groningen, Groningen, The Netherlands; 3 Department of Pathology and Medical Biology, University of Groningen, University Medical Center Groningen, Groningen, The Netherlands; 4 Department of Epidemiology, University of Groningen, University Medical Center Groningen, Groningen, The Netherlands

## Past

Oncological surgery is the cornerstone of treatment for gastrointestinal malignancies. Despite continuous improvement of surgical techniques and perioperative care, a substantial number of patients experience postoperative complications. To prevent these complications, many studies have focused on predictors of postoperative outcomes. These predictors also can help to improve patient selection for oncological surgery and therefore often are incorporated into the risk profile of a patient. Thus far, well-known factors include comorbidities, sarcopenia, and obesity.^1^ Formatting...Recently, systemic inflammation also has been found to be a predictor of complications, as evidenced by elevated levels of inflammatory cytokines, such as IL-6, IL-8, and IL-10.^2^

## Present

Inflammation and ischemia are intimately associated with oxidative stress. Therefore, markers of oxidative stress may constitute potential predictors of postoperative complications. One such marker is the serum free thiol level, the body’s most important nonenzymatic antioxidant, which may serve as a read-out of a whole-body redox status. Current studies describe that patients with cancer have lower serum free thiol levels than healthy people.^3^ Furthermore, studies in patients with different types of malignancies showed that patients with lower serum free thiol levels, reflecting a higher load of reactive species, have a lower overall survival. In addition, patients with impaired daily functioning have lower free thiol levels.^3^

In our study, we showed that preoperatively low serum free thiols are associated with complications and a longer hospital stay in patients with gastrointestinal cancer.^4^ Furthermore, serum free thiol levels decrease immediately after surgery, demonstrating the oxidative stress following surgery. Following this, the degree of oxidative stress was higher in patients with more blood loss and a longer time of anesthesia.

## Future

Our study indicates that the serum free thiol level may be promising as a predictor for postoperative complications after oncological surgery. Future studies should focus on determining optimal cutoff values, so that the measurement of thiol levels may be integrated into the preoperative work up. This could become a valuable addition to the existing risk profile of a patient, making preoperative counseling and interventions more precisely.

Furthermore, it might be possible to positively influence the antioxidant capacity of oncological patients, because free thiols are amendable to therapeutic interventions. However, future studies have to enlighten the complexity of an individual’s redox status to tailor the intervention to the individual patient. This is of cardinal importance since ROS also has crucial physiologic functions.

To attenuate preoperative levels of oxidative stress, it might be useful to focus on modifiable factors like lifestyle and diet. This is in line with implementation of prehabilitation strategies, where patients are already increasingly asked to change their diet, quit smoking, and increase their physical activity. Smoking and a sedentary lifestyle are negatively correlated with serum free thiol levels and thus might be possible targets for interventions. Besides, diet or the administration of antioxidants might be an addition to preoperative interventions to prevent complications. One example of an antioxidant is *N*-acetylcysteine (NAC), which promotes the generation of endogenous glutathione, as well as H_2_S, which protects against oxidative stress as a scavenger of reactive species and by upregulating the antioxidant system. Its benefit has been shown in small cohorts of COVID-19 patients.^5^ However, further research is warranted to determine the efficacy, safety, and tolerability of these potential redox-modulating interventions.
